# Advances in Design and Development of *Lumi-Solve*: A Novel Drug-Eluting Photo-Angioplasty Device

**DOI:** 10.1007/s13239-023-00668-0

**Published:** 2023-05-10

**Authors:** Amarnath Sangeetha Menon, Igor Subasic de Azevedo, Kylie Choong, Dhruv Bhatnagar, Chen Wang, Pavel Sluka, David R. Chisholm, Paul Pasic, Helmut Thissen, Gopal Sama, Andrea Robinson, Andrew Rodda, Aldous Tria, Loren Spiegel, Anak Dharma, Harikrishnan Kaipananickal, Jun Okabe, Assam El-Osta, Simon Mountford, Philip Thompson, Anthony E. Dear

**Affiliations:** 1 Eastern Health Clinical School, Monash University, Melbourne, Australia; 2 LightOx Ltd, Newcastle Upon Tyne, UK; 3 CSIRO Biomedical Materials Translational Facility, Melbourne, Australia; 4 School of Chemistry, Monash University, Melbourne, Australia; 5 Monash Institute of Medical Engineering, Monash University, Melbourne, Australia; 6 Monash University Department of Diabetes, Monash University, Melbourne, Australia; 7 Monash Institute of Pharmaceutical Sciences, Monash University, Melbourne, Australia; 8 Department of Vascular Surgery, Eastern Health, Box Hill Hospital, Melbourne, Australia

**Keywords:** Drug eluting balloon catheter, Neointimal hyperplasia, Fibre-optic, Guide wire shadow, Photo-activation

## Abstract

**Purpose:**

The Lumi-Solve photo-angioplasty drug eluting balloon catheter (DEBc) may afford safety advantages over current DEBc. Lumi-Solve utilises the guidewire (GW) port and lumen to deliver fibre-optic UV365nm light to the angioplasty balloon which may be problematic. We explore and evaluate alternative Lumi-Solve design options to circumvent fibre-optic use of the GW port and lumen which may enhance efficacy and clinical utility.

**Methods:**

Effects of guidewire shadowing (GWS) on visible and UV365nm light transmission were evaluated and modelled in-silico. To evaluate the effect of a dedicated intra-balloon fibre-optic port, modified angioplasty balloons and sections of translucent polyethylene terephthalate (PET) GW port tubing were utilised. Investigation of the effect of GWS on chemical and biological photo-activation of balloon surface drug was performed utilising LCMS analysis and inhibition of histone deacetylase activity (HDACi) was measured in human umbilical vein endothelial cells (HUVEC).

**Results:**

Parallel fibre-optic and GW port configurations generated a GWS of approximately 18.0% of the evaluable balloon surface area and attenuated both visible and UV light intensity by 20.0–25.0% and reduced chemical photo-activation of balloon surface drug and HDACi by at least 40–45%. Alternative fibre-optic port configurations including a spiral design significantly mitigated GWS effects on UV light transmission.

**Conclusions:**

To avoid use of the GW port and its associated complications a dedicated third port and lumen for the Lumi-Solve fibre-optic may be required. To maximize balloon surface chemical and biological photo-activation, non-parallel, intra-balloon, fibre-optic lumen trajectories, including a spiral design may be useful.

**Supplementary Information:**

The online version contains supplementary material available at 10.1007/s13239-023-00668-0.

## Introduction

We have recently described a novel drug-eluting, photo-activated balloon angioplasty device Lumi-Solve, for the management of post-angioplasty neointimal hyperplasia [1].

Current iterations of Lumi-Solve utilise guidewire (GW) port-mediated fibre-optic delivery of UV365nm light for balloon surface drug activation. GW port-mediated fibre-optic light delivery may be associated with issues including obstruction of light transmission due to GW port blood ingress, multiple insertions and removal of the GW and fibre optic and end-user concerns regarding intra-procedural GW removal. Design of a Lumi-Solve prototype housing a third, fibre-optic-dedicated port may circumvent these issues. Inherent in triple lumen catheter design is the potential for intra-balloon guidewire shadowing (GWS) which may impact Lumi-solve photo-activation.

GWS has been previously demonstrated to have significant effects in the context of vascular imaging and intravascular delivery of therapeutics [2, 3]. Previous design modifications aimed at attenuating the impact of the GWS have included alterations in GW composition [2, 4], adjustments aimed at increasing the distance from the GW thereby reducing shadow volume [3] together with intra-procedural GW removal [5].

To determine the potential significance of the GWS on Lumi-Solve photo-activation we first aimed to evaluate the effect of the GWS on angioplasty balloon surface light transmission using model systems and in-silico techniques. In addition, we aimed to evaluate and quantify the potential impact of GWS-mediated modulation of balloon surface light intensity, on both chemical and biological aspects of Lumi-Solve photo-activation and determine the contribution of alternate intra-balloon fibre-optic trajectories on these parameters.

## Methods

### In Silico Evaluation of GWS on Balloon Surface

ANSYS SPEOS optical system design software generated models of GWS intensity and area from prespecified balloon, GW and fibre-optic data quantifying potential GWS effects on balloon surface drug activation. A single fibre inside a 5.0 mm diameter balloon was used for the reference study. Additional assumed balloon dimensions include 30 mm length and 0.15 mm spacing between the fibre and the GW based on previous studies of catheter wall diameters [6] (Online Resource 1a). Balloon thickness is not included in simulation calculations as the inner wall is used as the irradiance study surface. Values of power delivery from the fibre were calculated based on information from the light source and fibre manufacturers to inform on irradiance levels [7–9]. Irradiance was measured as *W*atts/*m*etre^2^ (W/m^2^).

ANSYS SPEOS uses triangular mesh elements for surface irradiance studies (Online Resource 1b). Small absolute sag values (0.001 mm) are used to enhance mesh conformity and together with element sizing the ray tracing error is less than 0.0004% and the maximum lux error is 0.005% between repeated simulations. A light wavelength of 365 nm is selected with the GW and balloon set as opaque as the inner balloon wall is used as the irradiance surface. 10 million rays are traced with a geometric tolerance of 0.1 µm (Online Resource 1c). Higher resolutions showed insignificant improvement to error level with a much longer calculation time.

### Evaluation of GWS Utilising Intra-balloon UV Fibre-Optic Light Source

Standard Armada 35 PABA catheters, 4.0–5.0 mm diameter and 50.0–70.0 mm in length (Abbott Australasia Pty Ltd) and 0.035in, 150 cm GW (Boston Scientific, Starter™) were used together with customised 500 µm core fibre-optic, Model RD 30 with SMA 905 connector (Med*light* S.A, Switzerland), connected to a Silver-LED-365nmUV 100mW portable light source (Prizmatix, Israel) as previously described [1]. The Model RD 30 fibre-optic has a unique distal diffuser portion permitting uniform, radial transmission of UV365nm light along its entire length within the inflated angioplasty balloon [7].

To simulate an unobstructed, dedicated fibre-optic third lumen the fibre optic was introduced into the angioplasty balloon, via proximal and distal balloon punctures using a 19-gauge hypodermic needle. The activated fibre-optic was positioned 1.0–1.5 mm from the intra-balloon GW lumen (Online Resource 2). The GW was subsequently introduced and withdrawn from the intra-balloon space and images recorded.

### Evaluation of GWS on Balloon Surface UV365nm Area and Intensity

To evaluate the effect of GWS on angioplasty balloon surface light intensity and area we utilised a recently described UV365nm-activated, 500–650 nm emitting, fluorophore LightOx78 [10, 11] as a surrogate marker for direct UV365nm surface area coverage and intensity. LightOx78 was initially dissolved in DMSO and a 50:50% vol of 100% ethanol:Ultravist™ to give a 1.0 mmol/l final solution. A standard Armada 35 PABA catheter, 4.0–5.0 mm diameter and 60.0–70.0 mm in length (Abbott Australasia Pty Ltd) was ultrasonically coated, as previously described [12], with the LightOx78 1.0 mmol/l solution. The upper hemi-spherical aspect of the angioplasty balloon was then cleared of the LightOx78 coating to leave a lower hemi-spherically coated balloon for direct light-meter evaluation of fluorophore-generated visible light.

Equipment, including the LightOx78-coated Armada 35 PABA catheter, GW and intra-balloon fibre-optic (as outlined above) was arranged to permit detection of 500–650 nm wavelength light using a Radiometer RM12 light-meter with VISBG sensor (0–200 mW/cm^2^) (Opsytec Dr Gröbel, Germany). In addition, balloon ends were foil coated to limit fluorescent light detection to the region directly above the light sensor (Online Resource 3a-b). Subsequently, the Python™ computational analysis program was utilised to evaluate and quantify pixel brightness data from grey scale converted, superimposed (GW inserted and withdrawn) and rendered lightOX78-coated balloon images. Python™ analysis of these images enabled graphical presentation of the variability in balloon surface light intensity due to GWS.

To quantify balloon surface UV365nm intensity, an uncoated Armada 35 PABA with intra-balloon fibre-optic was arranged to permit UV365nm detection in the presence and absence of the GW using a Series 9811 365 nm Radiometer (Cole-Palmer Instrument Co, Chicago, ILL, USA light-meter (Online Resource 4a-c).

### Evaluation of Alternative Configuration of Fibre-Optic on GWS Effect

80–100 mm sections of translucent GW port were harvested from Armada 35 PABA catheters. Two sections of GW port where either (i) placed in parallel and fixed with surgical tape over a UV light-meter sensor or (ii) placed in a spiral configuration with one of the two sections of GW port spiralling around the other section which remained unaltered in configuration (Online Resource 5a-c). Spiral configurations ranging from 0.25 to 1.0 revolutions (low (0.25), medium (0.75) and high-density (1.0) coils) were evaluated for effect on GWS UV365nm transmission.

### Balloon Surface Activation of Caged-MCT-3

To quantify balloon surface photo-activation of caged-MCT-3 (c-MCT-3) an uncoated Armada 35 PABA with intra-balloon fibre-optic connected to UV365nm light source, as described above, was arranged to permit 4 min balloon surface UV365-mediated photo-activation of 1.3 mmol/l c-MCT-3 in the presence and absence of the GW (Online Resource 6). The activated samples (± GW) were obtained from the umbra GWS region of the balloon surface (Online resource 6 a and b) in order to optimize the potential impact of the GWS on chemical activation. Samples were subsequently used in liquid chromatography mass spectrometry (LCMS) and Western Blot analysis.

### High Performance Liquid Chromatography (HPLC) Analysis

#### HPLC Instrumentation and Conditions

HPLC analysis of c-MCT-3 and MCT-3 has been previously described [13]. Briefly, analysis utilised Agilent technologies 1220 infinity LC, equipped with diode array detector, vacuum degasser, automatic thermostatic column oven, a binary pump and computer with Agilent technologies 1220 infinity LC software for data analysis. 0.1% trifluoroacetic acid (TFA) in water and acetonitrile, an organic solvent, were used as mobile phase starting from 10% acetonitrile to 50% acetonitrile in 15 min and reaching initial concentration in 20 min. The analytical column (Waters Nova-Pack C_18,_ 3.9 × 150 mm) was equilibrated and eluted with mobile phase under gradient conditions with 1.2 ml/min flow rate at 30 °C column temperature. Sample detection was set at either 214 or 254 nm. Specificity of analyte analysis was confirmed with readily identifiable and complete peak separation demonstrated on HPLC tracings.

#### Preparation of Standard Solutions

10 µl of the 1.3 mmol/l standard solutions (c-MCT-3 and MCT-3) ± UV365nm activated samples were diluted to 1.0 ml with diluent (0.1% TFA in water and acetonitrile) and 25-100 µl was injected for analysis.

### In Vitro Studies

#### Cell Culture

Human umbilical vein endothelial cells (HUVECs) (CC-2519; Lonza, Switzerland)) were maintained in EGM-2 medium (Lonza) supplemented with penicillin/streptomycin, 10% fetal calf serum (FCS) at 37 °C in a 5% CO_2_ incubator.

The agents c-MCT-3 ± UV, ± GW were added to plates for 6 or 15 h at a final concentration of 10.0 μM.

### Determination of HDAC Activity by Western Blot

HUVECs treated with c-MCT-3 ± UV, ± GW or vehicle (DMSO) were washed with phosphate-buffered saline (PBS) and harvested using a cell scraper. Histones were isolated by acid extraction as previously described [13]. Protein concentrations were determined using Coomassie Reagent (Sigma) with BSA as a standard. Equal amounts (2 µg) of acid extract were separated by NuPAGE (Invitrogen, MA), transferred to a PVDF membrane (Immobilon-FL; Millipore) and then probed with antibodies against H3K9/14ac (06-599, Millipore) and total histone H3 (#14269; Cell Signaling Technology, MA). Protein blotting signals were quantified by an infrared imaging system (Odyssey CLx image system; LI-COR Biotechnology, NE). H3K9/14ac levels were quantified using total histone H3 signal as a loading control.

### Statistical Analysis

Results were expressed as means ± standard error of the mean (SEM), and analysed using GraphPad Prism 5 software, using unpaired *t* tests for two-group comparisons and one-way analysis of variance (ANOVA) followed by Tukey’s post hoc ANOVA for three or more group comparisons. *P* value of < 0.05 was considered statistically significant.

## Results

### In-Silico Evaluation of GWS Effect

**Fig. 1:**
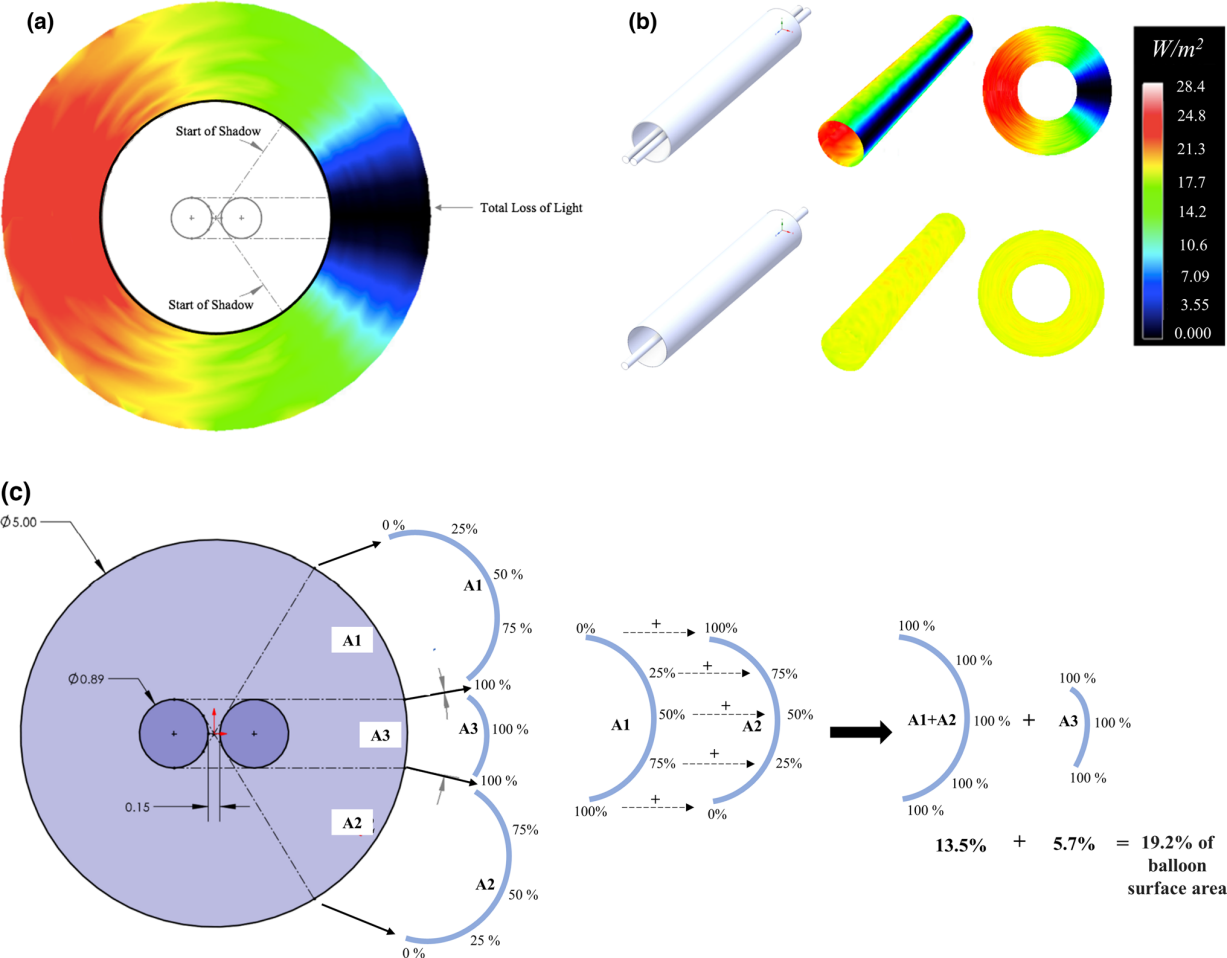
**a** and **b** ANSYS-SPEOS cross-sectional (**a**) and longitudinal (**b**) quantitative (W/m^2^) representation of effect of GWS on balloon surface light radiance, fibre-optic on the left, GW on the right arranged in parallel. Lower panel represents fibre-optic alone balloon surface radiance quantitation. **c** ANSYS-SPEOS evaluation of GWS area on balloon surface. A_3_ = 5.7% of balloon area in 100% light loss. A_1_ = A_2_ = 13.5% of balloon surface area with graded light loss (0–100%). A_1_ + A_2_ + A_3_ = 32.7% balloon surface under some shadow and (A_1_ + A_2_) + A_3_ = (13.5%) + 5.7% = 19.2% balloon surface experiences the equivalent of 100% light loss

ANSYS-SPEOS light simulation programme analysis of a 5.0 mm diameter Armada 35 angioplasty balloon with parallel guidewire and fibre-optic lumens (Fig. 1a, b) identified balloon surface area impacted by the GWS of 32.7%, with 19.2% effectively receiving no light (Fig. 1c). Increased surface radiance nearest to the fibre-optic (Fig. 1a) over fibre alone surface radiance (Fig. 1b, lower panel) reflects the closer proximity of the fibre optic to the balloon wall in the parallel GW, fibre optic orientation.

Additional reflectance and absorption modelling studies were performed. Reflectance studies utilising previously published polyethylene terephthalate (PET) reflectance values [14] demonstrated a nominal (≤ 1.5%) increase in UV365nm light intensity for parallel orientation of guidewire and UV fibre-optic consistent with the UV365nm transmission spectra of PET [15] (data not shown). Absorbance studies utilising previously published polytetrafluoroethylene (PTFE) absorbance values [16], representative of the standard guidewire coating, also demonstrated a nominal (≤ 2.0%) change in maximum UV365nm light intensity for the parallel GW/fibre-optic configuration (data not shown).

**Fig. 2:**
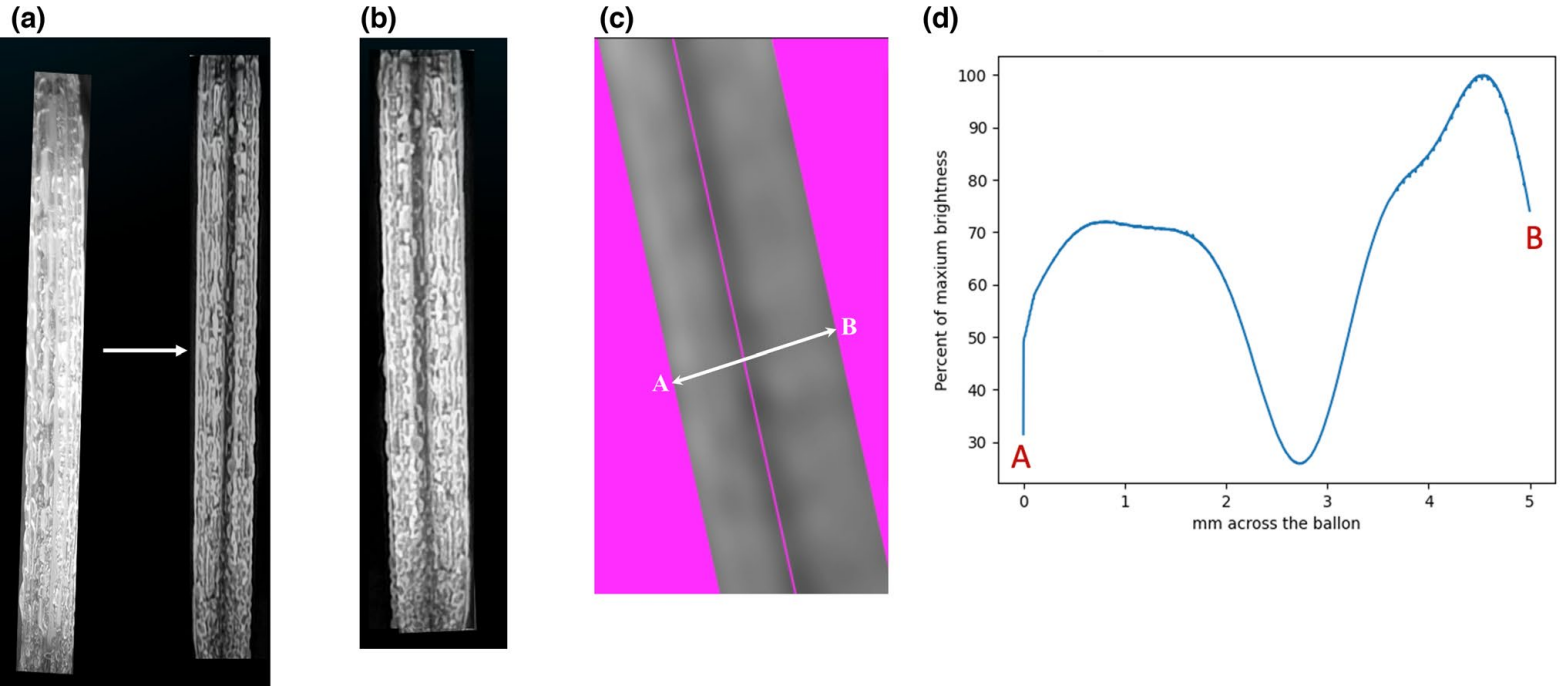
**a**–**d** Python program pixel analysis. **a** Grey scale images of LightOx78 coated angioplasty balloon in the absence (left) and presence (right) of 0.89in GW. Arrow indicates images are superimposed to generate image (**b**) which is subsequently rendered and pixel evaluation calculated between points A and B (**c**). Graphical presentation of area and reduction in light intensity effect of GWS on balloon surface (**d**)

Python™ analysis of a rendered image from a 5.0 mm diameter Armada 35 angioplasty balloon identified balloon surface area impacted by the GWS (Fig. 2a–c). The area of balloon affected by the GWS was observed from 1.8 mm and extended to 3.2 mm across the 5.0 mm balloon diameter and identified a 40% reduction in maximum brightness (70% ~ decreasing to 30% ~) (Fig. 2d). Average balloon surface area impacted by GWS, as derived by Python™ analysis, was determined to be approximately 17.6% of the total balloon surface area evaluated from the images utilised for analysis (Fig. 2c).

### Quantification of GWS Effect on Balloon Surface Light Intensity

**Fig. 3:**
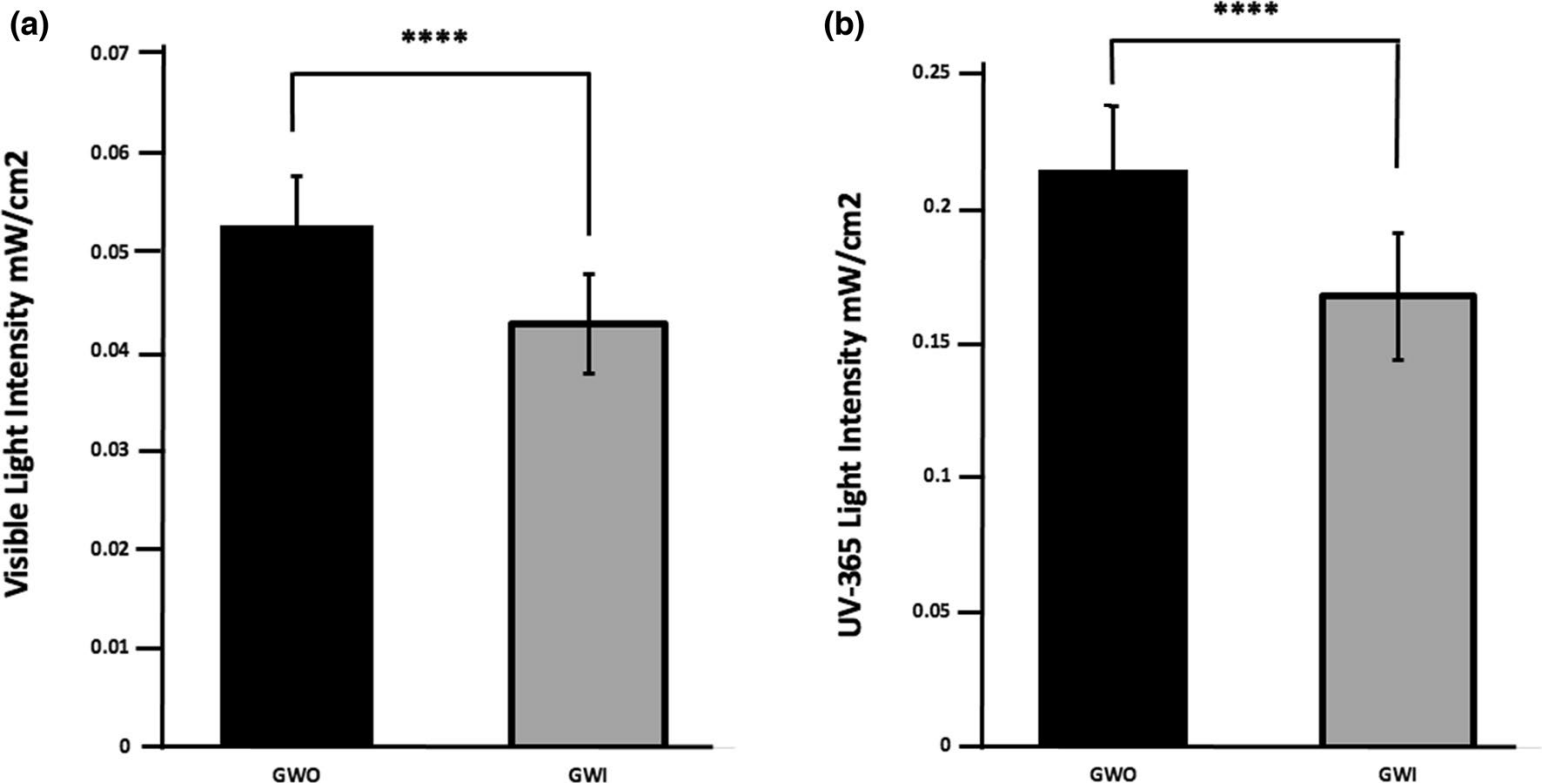
**a** 500–650 nm green/yellow visible light detection and GW position. 500–650 nm detection (m*W*/cm^2^) with GW fully removed (GWO) or inserted (GWI) from angioplasty balloon. ****p < 0.0001 GWO vs. GWI, n = 20 passes. **b** 365 nm UV light detection and GW position. 365 nm UV detection (m*W*/cm^2^) with GW fully removed (GWO) or inserted (GWI) from angioplasty balloon. ****p < 0.0001 GWO vs. GWI, n = 32 passes

GWS significantly attenuated the intensity of white light transmission to the balloon surface with a reduction of approximately 20% in visible light intensity observed (Fig. 3a).

Specific characterisation of GWS-mediated attenuation of UV365nm light transmission to balloon surface also demonstrated a significant, approximately 20% reduction in UV365nm light transmission (Fig. 3b).

### GWS Effect on Chemical and Biological Activation of Balloon Surface c-MCT-3

**Fig. 4:**
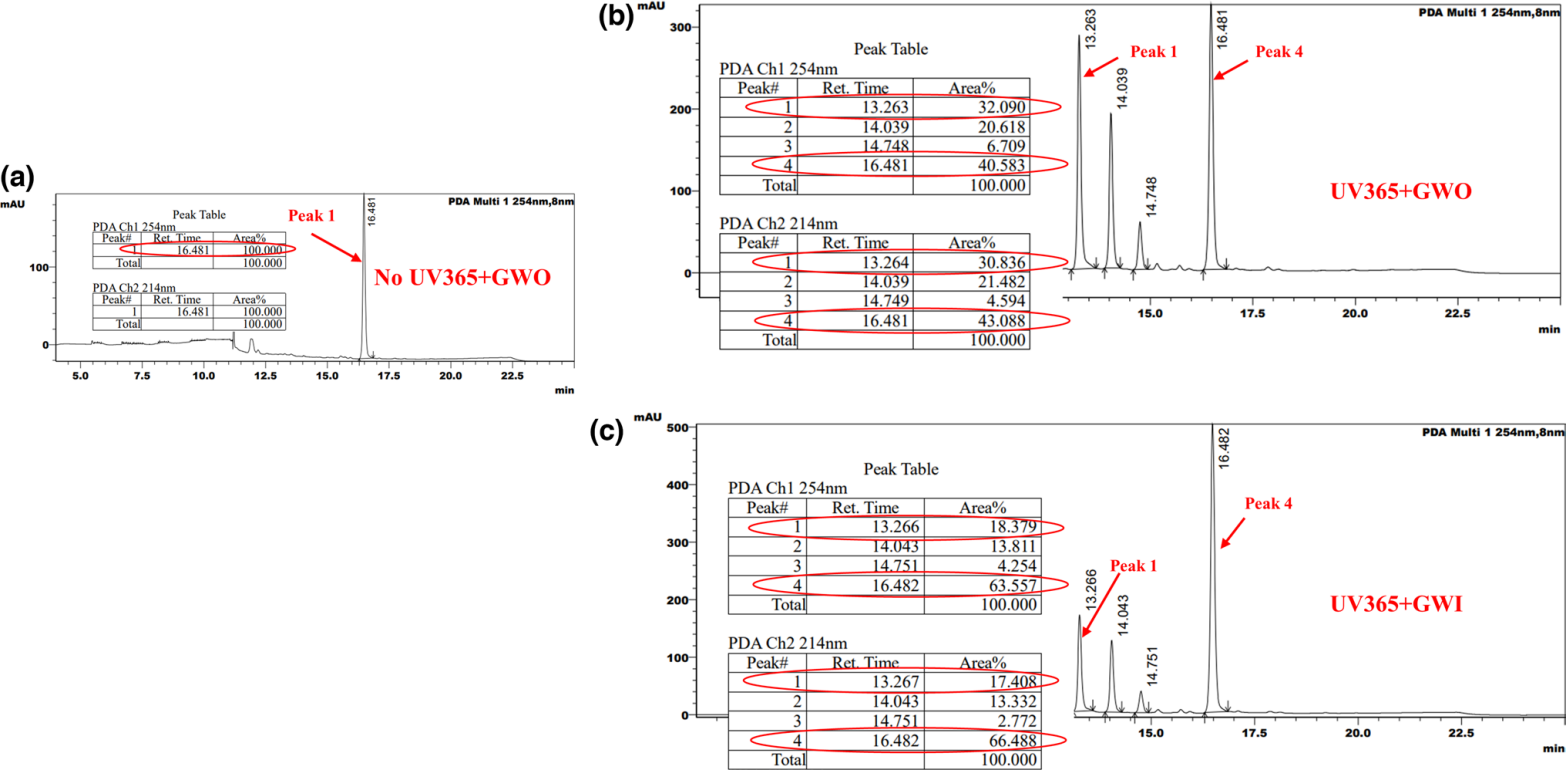
HPLC analysis of conversion of c-MCT-3 to MCT-3 at 254 and 214 nm **a** HPLC trace with no UV365nm exposure. c-MCT-3 peak #1. **b** HPLC from 1.3 mmol/L c-MCT-3 with 4 min UV365nm exposure, GW out (GWO). c-MCT-3 peak #4, MCT-3, peak #1 and **c** 1.3 mmol/L c-MCT-3 with 4 min UV365nm exposure, GW in (GWI). c-MCT-3, peak #4, MCT-3, peak #1

HPLC analysis (254 and 214 nm) of c-MCT-3 activation on angioplasty balloon surface with no UV and no GW (GWO) demonstrated no conversion of c-MCT-3 to MCT-3 (Online Resource 6) (Fig. 4a). 4 min of UV365nm activation together with no GW present (GWO) demonstrated 32.1% conversion to MCT-3 (Fig. 4b). 4 min of UV365nm activation together with insertion of the GW (GWI) resulted in an approximate 45% reduction in c-MCT-3 activation with 18.4% conversion of c-MCT-3 to MCT-3 (Fig. 4c).

**Fig. 5:**
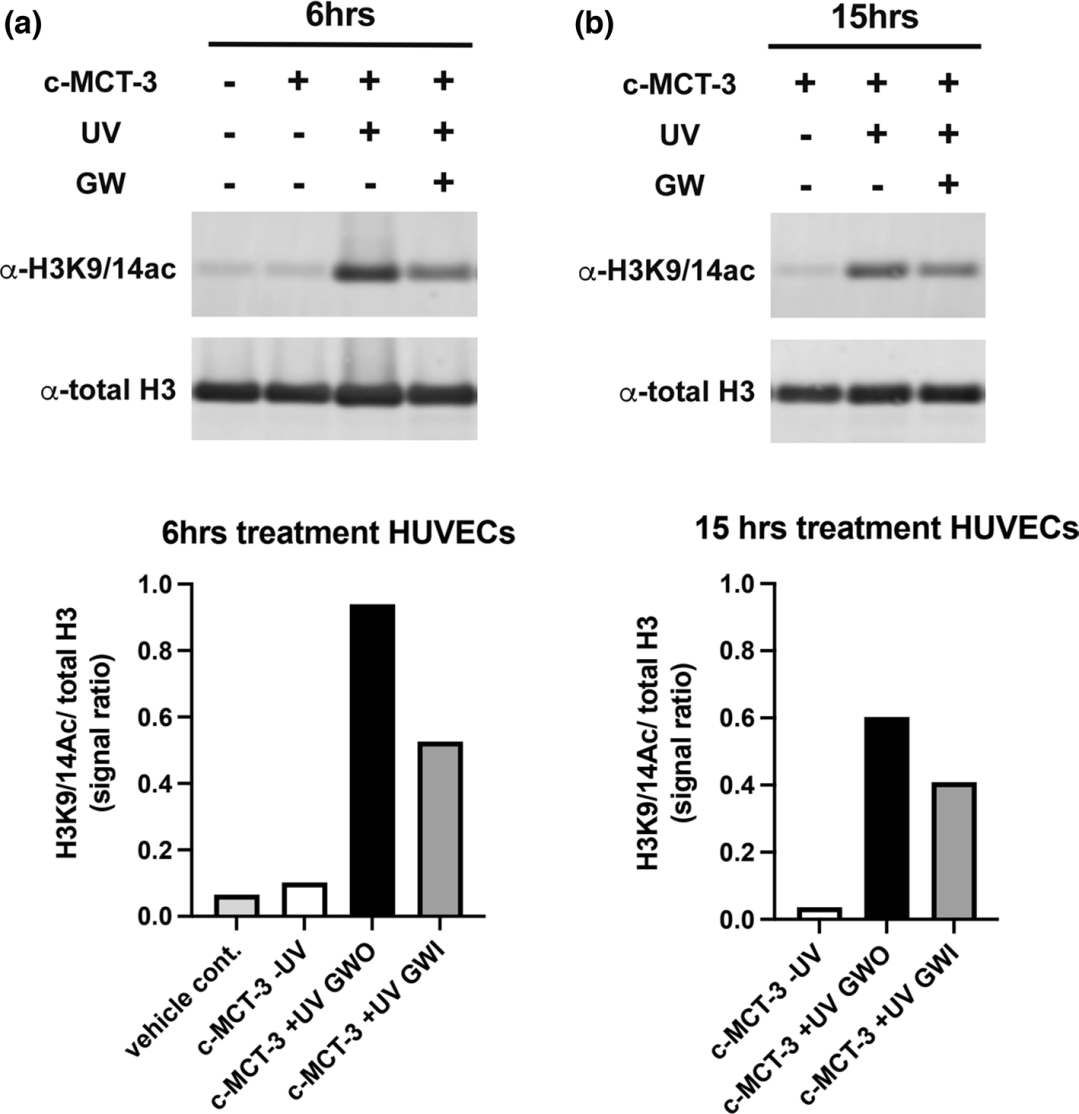
GWS effect on HDACi in human umbilical vein endothelial cells (HUVECs). Acetylation of histone H3K9/14 (H3K9/14ac) level was quantified in HUVEC by the LI-COR Odyssey assay. The cells were treated with 10 µM of c-MCT-3 ± UV365nm, ± GW inserted for 6 (**a**) or 15 (**b**) hours

Biological evaluation of HDACi in HUVEC from GWO and GWI balloon surface samples activated with UV365nm for 4 min demonstrated a significant attenuation in the level of H3K9/14 acetylation in GWI samples at 6 (50% reduction) and 15 h (30% reduction) (Fig. 5a, b) suggesting that GW-mediated reduction in UV365nm activation of c-MCT-3 to MCT-3 (Fig. 4) translates into reduced HDACi-mediated biological activity.

Targeting of c-MCT-3 droplets to within the GW shadow umbra region A3 (Fig. 1c) correlates with the 45–50% reduction in UV365nm-mediated conversion of c-MCT-3 and reduced HDACi activity as would be expected in an area of total light absence. As PET, the principal material utilized in the manufacture of PABAs, has previously demonstrated significant UV365nm transmission [15], the contribution of internal balloon light reflectance of UV365nm light to c-MCT-3 activation is considered minimal.

### Evaluation of an Alternative Fibre-Optic Configuration on GWS Effect on UV365nm Intensity

**Fig. 6:**
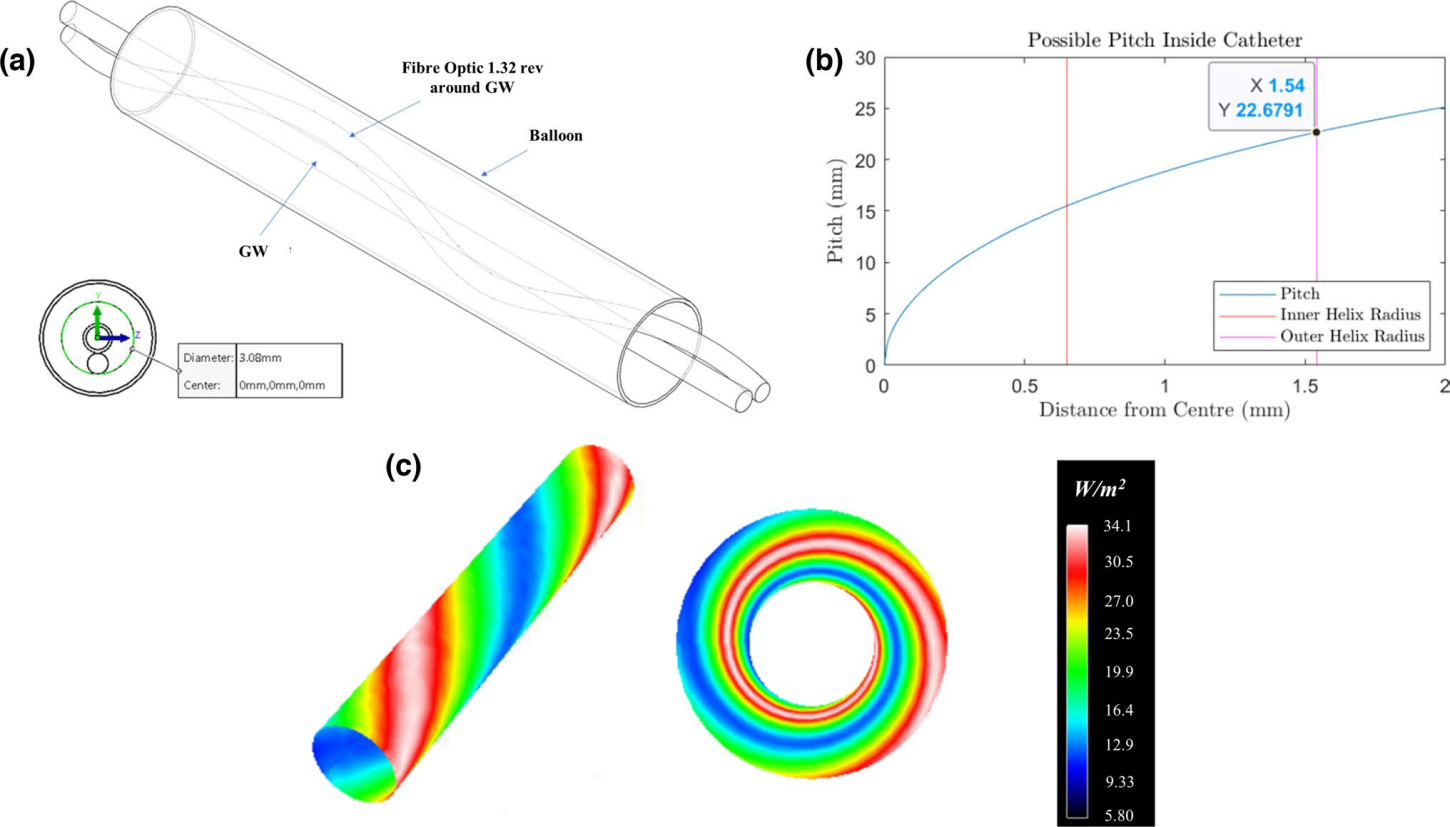
**a** Illustration of coiling concept of fibre optic around GW port in a 30.0 mm balloon. The outer helix diameter (see insert, bottom left) identifies a catheter with a diameter of greater than 3.0 mm. **b** Plot of helix pitch vs. distance from centre of catheter assuming a minimum fibre-optic radius of curvature of 10.0 mm. For a 30.0 mm balloon, a pitch of 22.6791 mm results in ~ 1.32 fibre-optic revolutions around the central GW port. **c** ANSYS-SPEOS quantitative contour plot of irradiance for coiled fibre-optic longitudinally and in cross-section

Potential alternative intra-balloon fibre-optic trajectories may produce less GWS effects. ANSYS SPEOS evaluation of a spiral fibre-optic configuration around a central GW lumen identified a maximum of 1.32 fibre-optic revolutions, given pitch and radius of curvature constraints, in the context of a 30.0 mm long balloon, resulting in an improved balloon surface radiance over parallel GW and fibre-optic trajectories. However, significant light variance was identified with areas of 18% of maximum radiance noted together with a minimum catheter diameter requirement of 3.0 mm necessary to accommodate pitch requirements of the design (Fig. 6a–c). Additional reflectance modelling studies were performed utilising PET reflectance values [14]. The spiral UV-fibre-optic orientation identified a marginal (~ 4.0%) increase in maximum UV365nm light intensity (data not shown). Absorbance studies utilising polytetrafluoroethylene (PTFE) absorbance values [15], demonstrated a ≤ 2.0% change in maximum UV365nm light intensity for the spiral configuration.

**Fig. 7:**
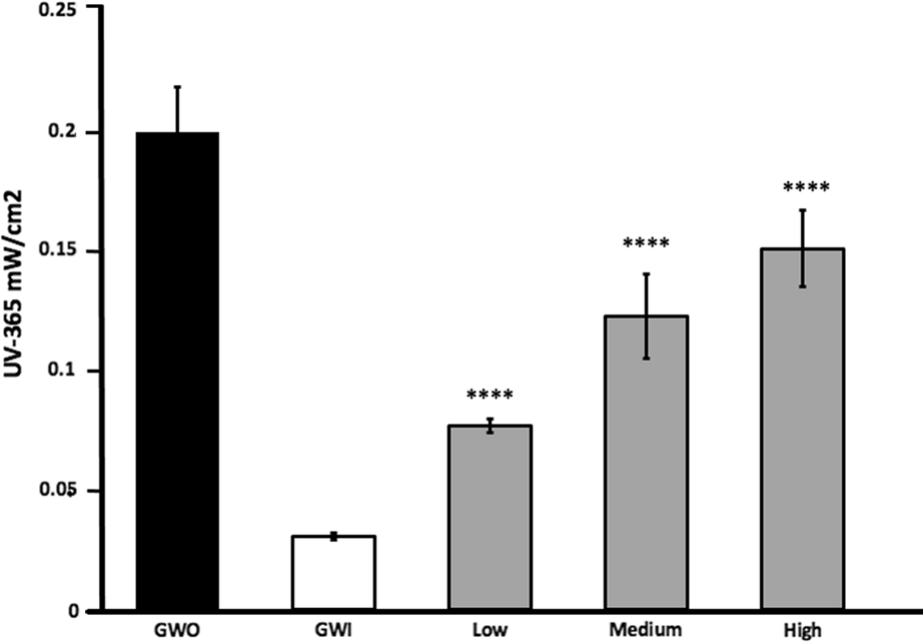
Spiral fibre-optic trajectory and quantification of UV365nm light detection. UV365nm detection (m*W*/cm^2^) with GW out (GWO), GW in (GWI) and low, medium and high, fibre-optic coil density models. GWI vs. low, medium, high, ****p < 0.0001, n = 6–12 passes

To determine the potential of a circumferential spiral fibre-optic configuration to attenuate the GWS effect on UV365nm light transmission a series of spirals with increasing coil density were evaluated (Online Resource 5). As the Med*light* S.A cylindrical light diffuser model RD used in our studies delivers uniform, radial light over the entire distal, intra-balloon, portion of the fibre-optic the only constraints to spiralling the distal diffuser component, and potential for light losses, reside in a prespecified minimum bending radius of 10.0 mm as per manufacturers specifications [7]. GWS attenuated UV light transmission by 87.5%, permitting only 12.5% UV365nm light transmission (GWI) compared with GWO (Fig. 7). Spirals with low, medium and high coil density significantly restored UV365nm light transmission permitting up to 42%, 78% and 94% UV365nm light transmission respectively (Fig. 7). The increased GWS effect on attenuation of UV light transmission compared with that identified in Fig. 3 reflected the closer proximity of the GW to the fibre-optic (Online Resource 2 vs 5) resulting in a denser shadow umbra and area as previously demonstrated [17]. To characterise the impact of fibre coiling on chemical activation of c-MCT-3, maximum power output from the Prizmatix portable UV365nm light source was reduced to 90% to simulate the effect of high density fibre coiling (Fig. 7). HPLC analysis of balloon surface c-MCT-3 activation under these conditions yielded no significant loss of activation of c-MCT-3 to the active agent MCT-3 (data not shown).

A spiral fibre-optic port configuration demonstrated significantly improved balloon UV radiance although was still impacted by the effect of GWS.

## Discussion

Lumi-Solve affords a significant advance in DEBc technology offering a targeted and potentially safer alternative to currently available DEB’c. Current iterations of Lumi-Solve utilise the guidewire port to deliver fibre-optic-mediated UV365nm light to the c-MCT-3 coated balloon facilitating device activation, an inherently problematic procedure, with associated efficacy, safety and clinical utility considerations. A dedicated third port, triple lumen-based, catheter design may circumvent the problems associated with the guidewire port fibre-optic delivery strategy currently utilised. A blind-ending dedicated third port for fibre-optic passage, by virtue of its capacity to insulate the fibre-optic from all contact with the intra-vascular space, may also obviate the single-use status currently ascribed to the Lumi-Solve fibre-optic, hence significantly reducing the economic burden currently associated with this endovascular intervention.

In considering a dedicated fibre-optic port Lumi-Solve design, concerns regarding the potential impact of GWS on balloon surface photo-activation are identified with significant GWS created and concomitant attenuation of balloon surface UV365nm light transmission and chemical and biological activation of c-MCT-3 observed. Limitations of our observations include the potential effects of material absorptivity, reflectivity and transmissivity together with light attenuation due to fibre length although these have been judged to be insignificant based on our in-silico evaluation studies.

The demonstration of a significant GWS in our studies suggests a potential for obstruction to balloon surface, c-MCT-3 photo-activation, and hence delivery of adequate active drug to the vessel surface. The impact of GWS and the need to resolve this issue in clinical scenarios including intravascular imaging [2, 4, 5, 18, 19] demonstrates the clinical significance of GWS and potential relevance to Lumi-Solve photo-activation. Identification of GWS-mediated attenuation of intra-vascular brachytherapy for restenosis by approximately 70% [3] together with our observation of a 45% reduction in activation of c-MCT-3 due to the presence of GWS highlight the significance of this effect (Figs. 4, 5). In addition, previous studies utilising HDACi have identified a correlation between the dose dependent effect on histone acetylation and inhibition of vascular smooth muscle cell proliferation, suggesting that GWS-mediated attenuation of c-MCT-3 biological activation may have a significant impact on clinical efficacy of our Lumi-Solve device [20, 21].

In designing potential alternative intra-balloon fibre-optic trajectories to circumvent GWS a spiral or coiled configuration may represent a solution which our preliminary data supports. Early design concepts in relation to laser atherectomy catheters recognised the potential utility of a spiral/coiled intra-balloon portion of the laser fibre in order to avert potential GWS [22]. Subjecting the spiral fibre-optic trajectory to in-silico analysis identified improved balloon radiance potential however some impact from GWS remained. Akin to laser atherectomy catheter design advances a multi-fibre-optic design may eliminate any residual GWS effects and could represent a more advanced solution.

Whilst the clinical significance of GWS in the context of photo-activated balloon angioplasty has not been previously explored our in-silico and in-vitro studies provide compelling evidence that GWS will mediate attenuation of balloon surface drug activation and hence efficacy of our Lumi-Solve device, necessitating the current exploration of alternative intra-balloon fibre-optic designs. Our ongoing studies will aim to evaluate balloon surface photo-activation in prototype Lumi-Solve devices manufactured with a dedicated third lumen and intra-balloon optic-fibre designs aimed at eliminating the potential effects of GWS on device activation and efficacy.

## Supplementary Information

Below is the link to the electronic supplementary material.

Supplementary file1 **Online Resource 1 (ESM_1)** Images (a-c) of ANSYS SPEOS light simulation modelling. (PPTX 786 kb)

Supplementary file2 **Online Resource 2 (ESM_2)** Image of triple lumen catheter model with intra-balloon fibre -optic. (PPTX 2166 kb)

Supplementary file3 **Online Resource 3 (ESM_3) 3a** Apparatus for detection of balloon surface visible light. (PPTX 1480 kb)

Supplementary file4 **Online Resource 3 (ESM_3) 3b-c** Magnification and animation of apparatus for detection of balloon surface visible light. (PPTX 14361 kb)

Supplementary file5 **Online Resource 4 (ESM_4) 4a** Apparatus for detection of balloon surface UV365nm light. (PPTX 2112 kb)

Supplementary file6 **Online Resource 4 (ESM_4) 4b** Magnification of apparatus for detection of balloon surface UV365nm light. (PPTX 1083 kb)

Supplementary file7 **Online Resource 4 (ESM_4) 4c** Demonstration of GW impact on UV365nm light transmission. (PPTX 55415 kb)

Supplementary file8 **Online Resource 5 (ESM_5) 5a-c** Apparatus for determination of parallel vs. spiral fibre-optic orientation on UV365nm light transmission. (PPTX 8704 kb)

Supplementary file9 **Online Resource 6 (ESM_6) 6a-b**. Apparatus, demonstration and image GW effect on balloon surface c-MCT-3 activation. (PPTX 14238 kb)

## Data Availability

All data generated or analyzed during this study are included in this manuscript and its supplementary information files.
