# Supplementary figures and images for: Advances in Design and Development of Lumi-Solve: A Novel Drug-Eluting Photo-Angioplasty Device
Source: Cardiovasc Eng Technol. 2023 May 10;14(4):605–14. doi: 10.1007/s13239-023-00668-0 (PMC10465377; doi:10.1007/s13239-023-00668-0)

## Slide 1
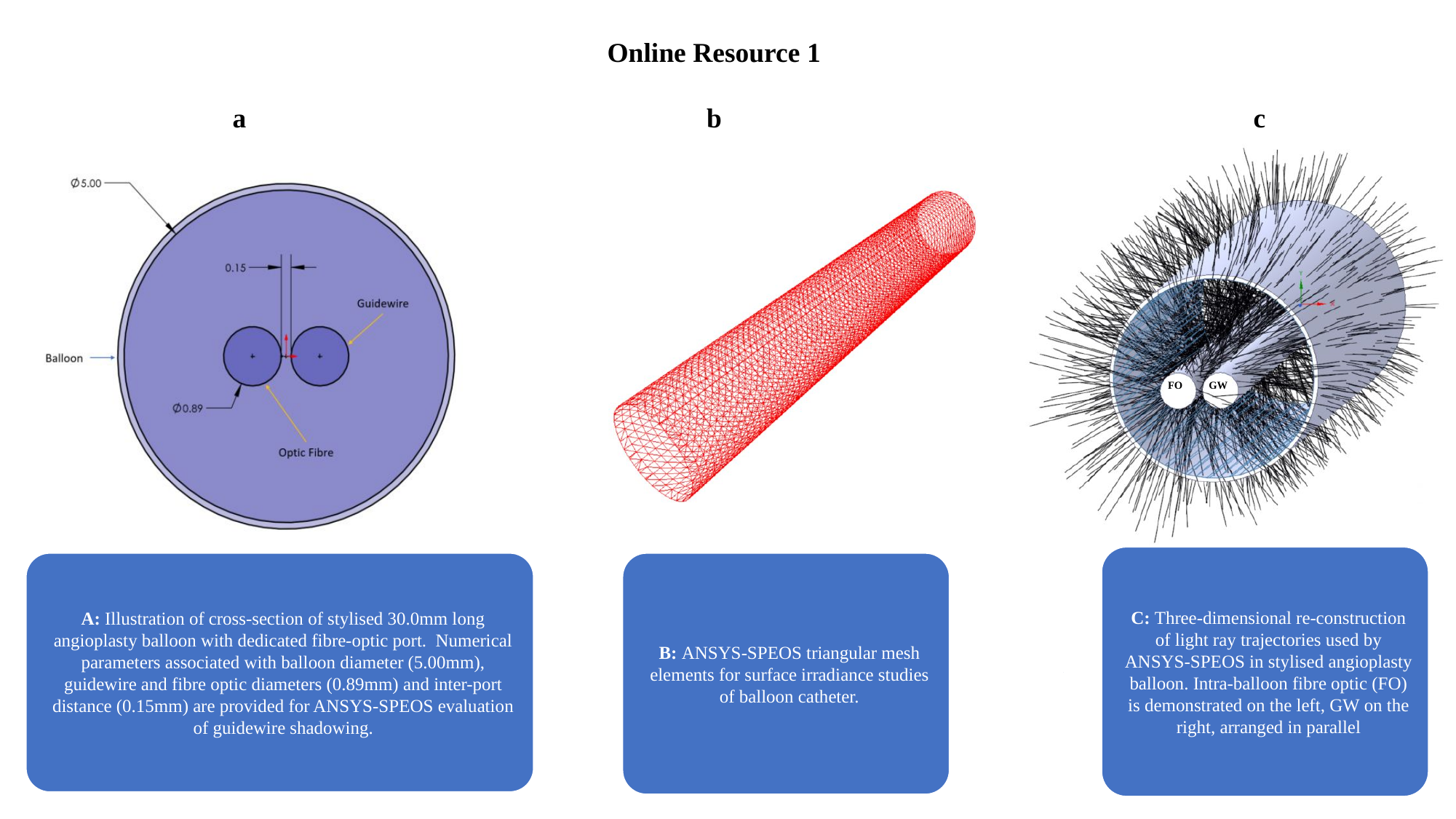

Online Resource 1
c
a
b
GW
FO

Supplement: Supplementary file 1 — Supplementary file1 Online Resource 1 (ESM_1) Images (a-c) of ANSYS SPEOS light simulation modelling. (PPTX 786 kb) [file 13239_2023_668_MOESM1_ESM.pptx]

## Slide 1
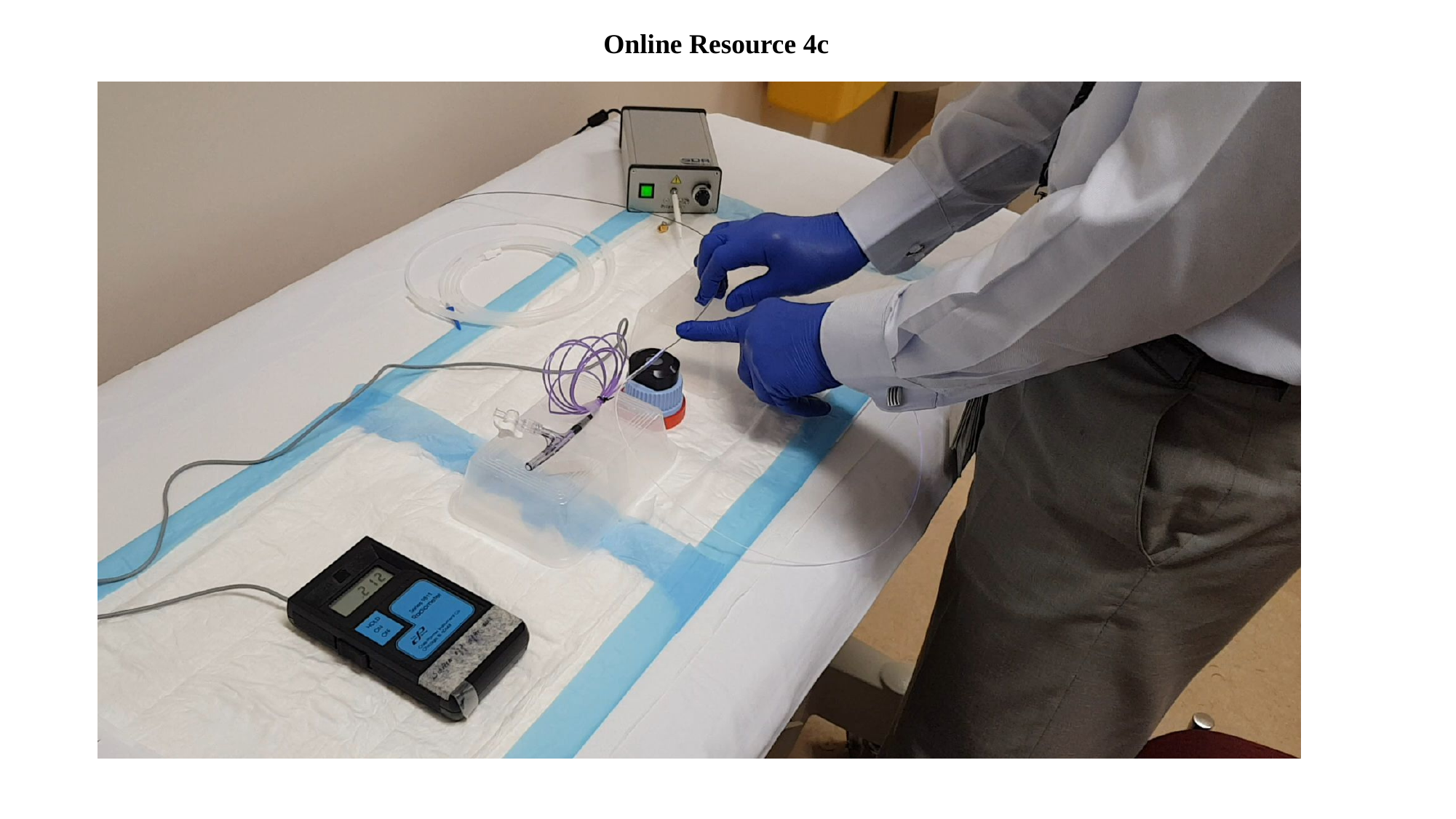

Online Resource 4c

Supplement: Supplementary file 7 — Supplementary file7 Online Resource 4 (ESM_4) 4c Demonstration of GW impact on UV365nm light transmission. (PPTX 55415 kb) [file 13239_2023_668_MOESM7_ESM.pptx]
