# Supplementary material for: Advances in Design and Development of Lumi-Solve: A Novel Drug-Eluting Photo-Angioplasty Device
Source: Cardiovasc Eng Technol. 2023 May 10;14(4):605–14. doi: 10.1007/s13239-023-00668-0 (PMC10465377; doi:10.1007/s13239-023-00668-0)
Supplement: Supplementary file 2 — Supplementary file2 Online Resource 2 (ESM_2) Image of triple lumen catheter model with intra-balloon fibre -optic. (PPTX 2166 kb) [file 13239_2023_668_MOESM2_ESM.pptx]

## Slide 1
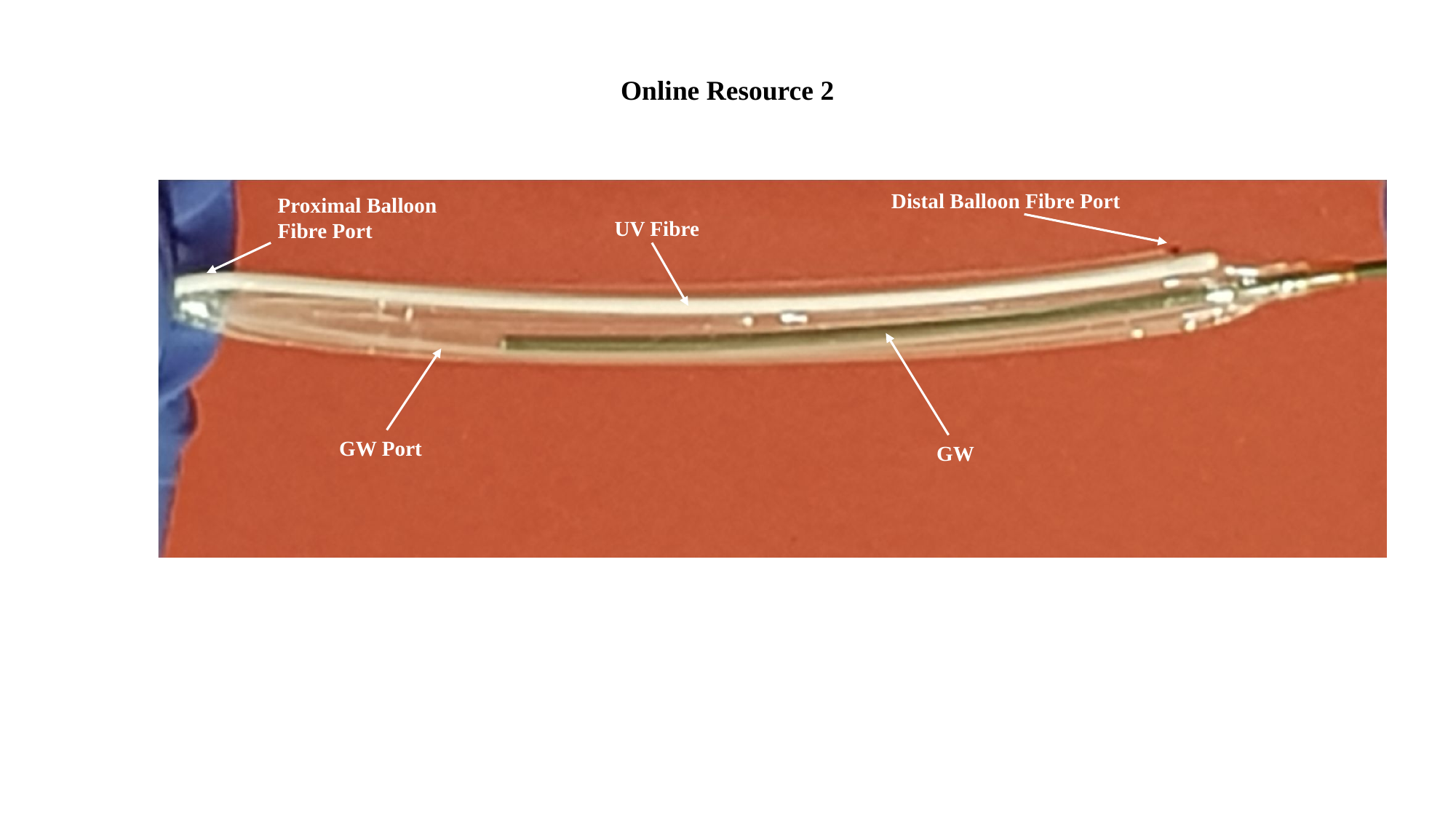

Online Resource 2
Distal Balloon Fibre Port
Proximal Balloon Fibre Port
UV Fibre
GW Port
GW
