# Supplementary material for: Advances in Design and Development of Lumi-Solve: A Novel Drug-Eluting Photo-Angioplasty Device
Source: Cardiovasc Eng Technol. 2023 May 10;14(4):605–14. doi: 10.1007/s13239-023-00668-0 (PMC10465377; doi:10.1007/s13239-023-00668-0)
Supplement: Supplementary file 3 — Supplementary file3 Online Resource 3 (ESM_3) 3a Apparatus for detection of balloon surface visible light. (PPTX 1480 kb) [file 13239_2023_668_MOESM3_ESM.pptx]

## Slide 1
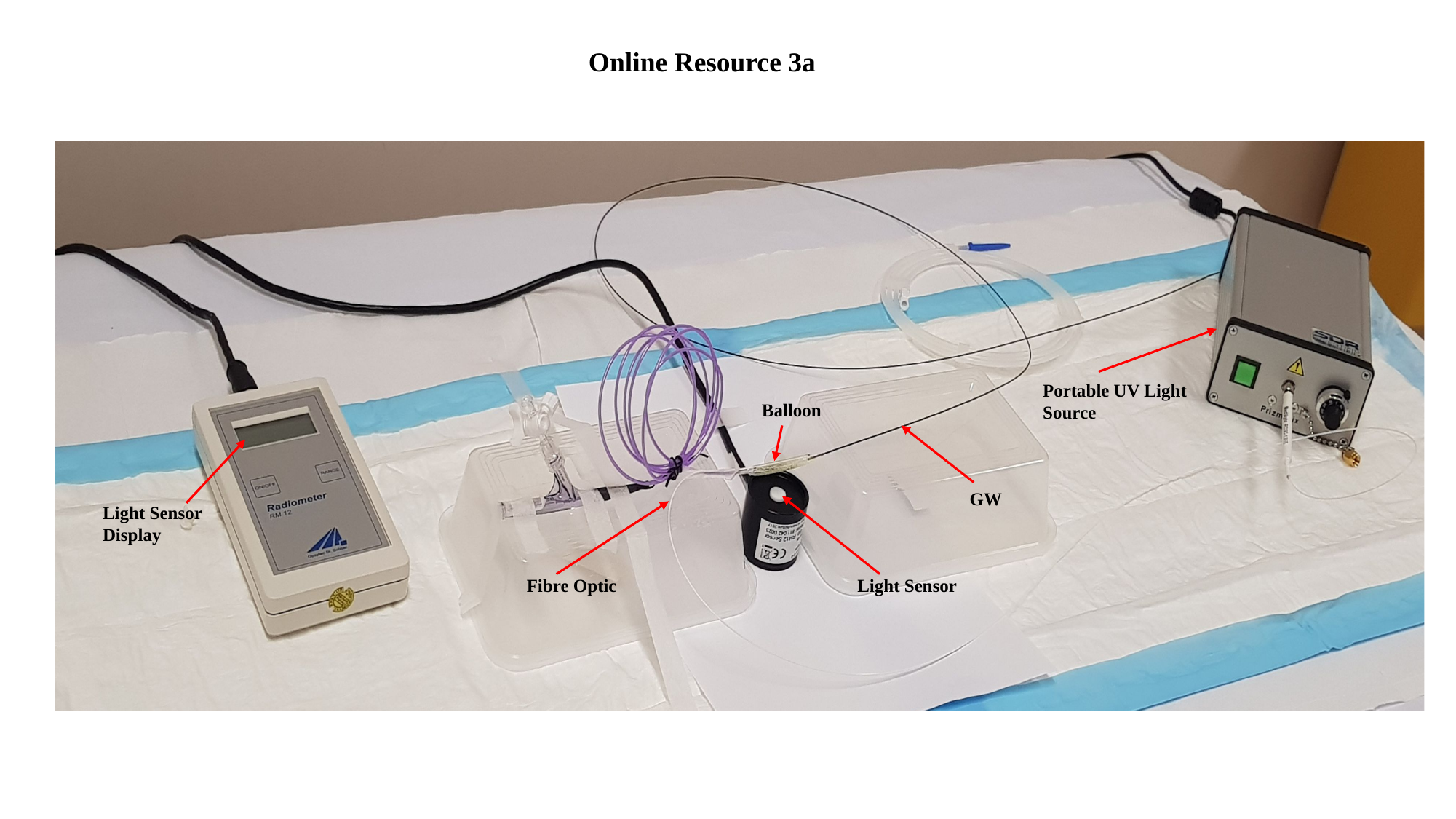

Online Resource 3a
#
Light Sensor (500-650nm)
Portable UV Light Source
Balloon
GW
Light Sensor Display
Light Sensor
Fibre Optic
