# Supplementary material for: Advances in Design and Development of Lumi-Solve: A Novel Drug-Eluting Photo-Angioplasty Device
Source: Cardiovasc Eng Technol. 2023 May 10;14(4):605–14. doi: 10.1007/s13239-023-00668-0 (PMC10465377; doi:10.1007/s13239-023-00668-0)
Supplement: Supplementary file 4 — Supplementary file4 Online Resource 3 (ESM_3) 3b-c Magnification and animation of apparatus for detection of balloon surface visible light. (PPTX 14361 kb) [file 13239_2023_668_MOESM4_ESM.pptx]

## Slide 1
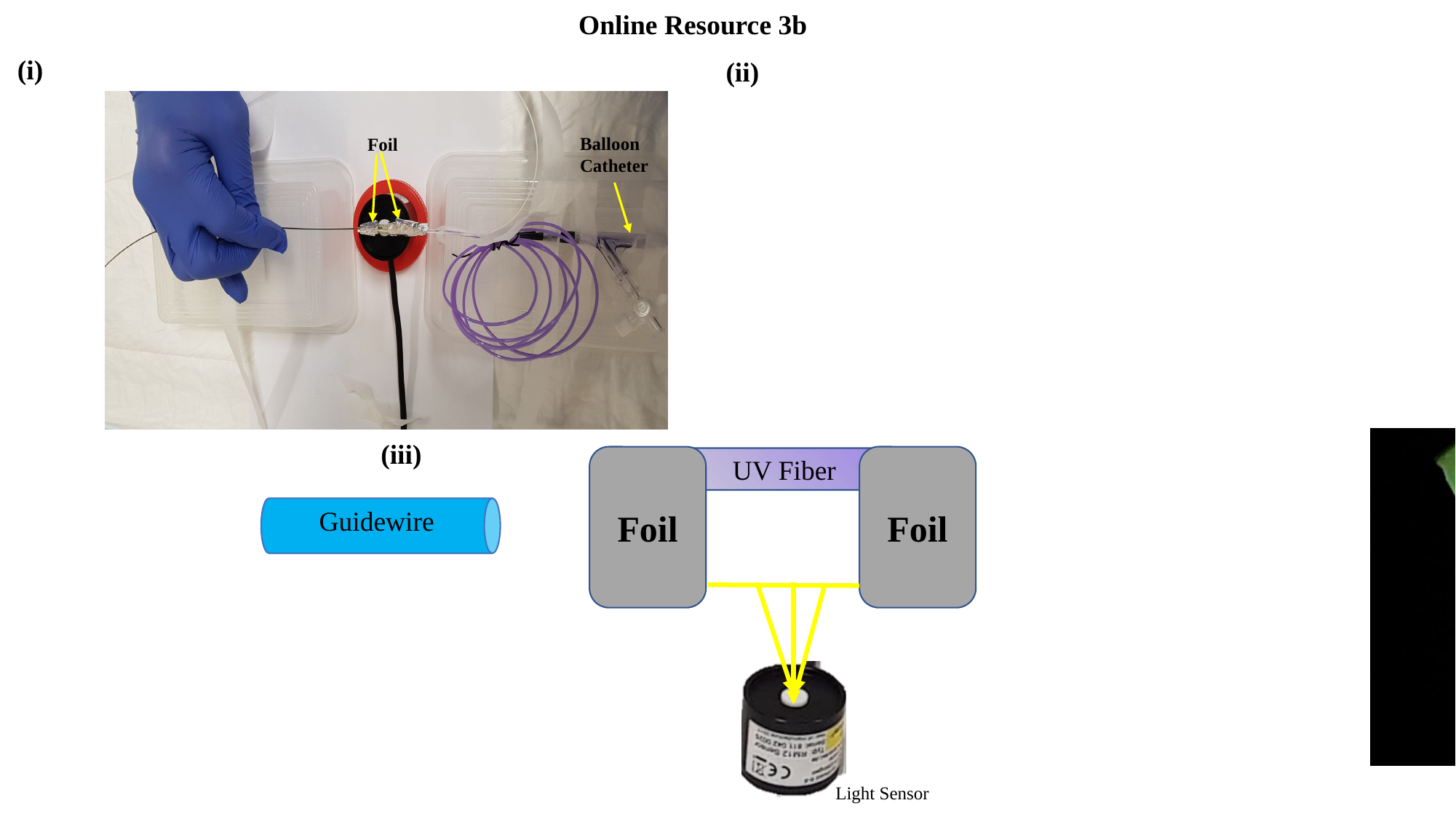

Online Resource 3b
(i)
(ii)
Foil
Balloon Catheter
 UV Fiber
Guidewire
(iii)
Foil
Foil
Light Sensor
