# Supplementary material for: Advances in Design and Development of Lumi-Solve: A Novel Drug-Eluting Photo-Angioplasty Device
Source: Cardiovasc Eng Technol. 2023 May 10;14(4):605–14. doi: 10.1007/s13239-023-00668-0 (PMC10465377; doi:10.1007/s13239-023-00668-0)
Supplement: Supplementary file 5 — Supplementary file5 Online Resource 4 (ESM_4) 4a Apparatus for detection of balloon surface UV365nm light. (PPTX 2112 kb) [file 13239_2023_668_MOESM5_ESM.pptx]

## Slide 1
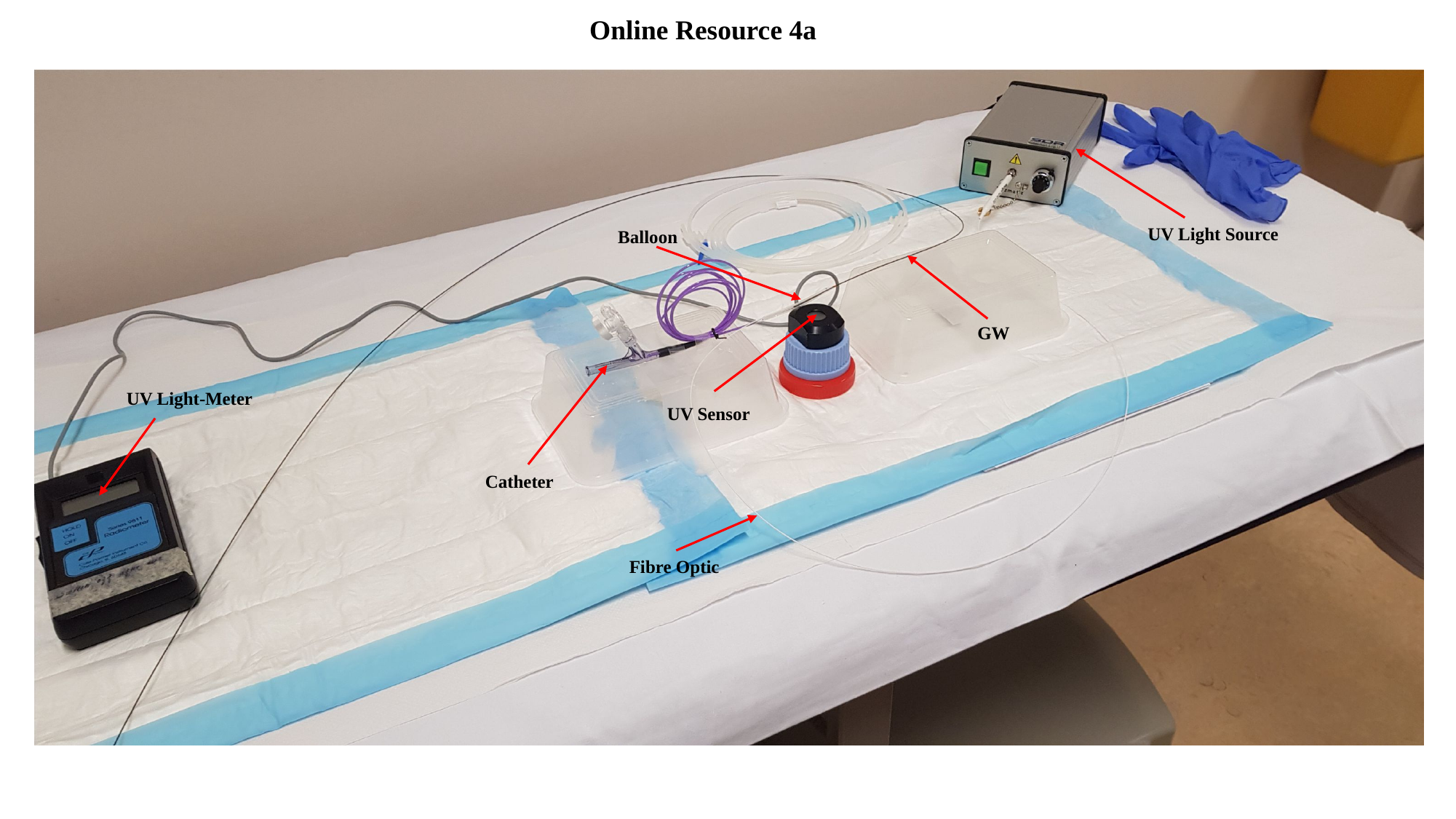

Online Resource 4a
#
UV Light Source
Balloon
GW
UV Light-Meter
UV Sensor
Catheter
Fibre Optic
