# Supplementary material for: Advances in Design and Development of Lumi-Solve: A Novel Drug-Eluting Photo-Angioplasty Device
Source: Cardiovasc Eng Technol. 2023 May 10;14(4):605–14. doi: 10.1007/s13239-023-00668-0 (PMC10465377; doi:10.1007/s13239-023-00668-0)
Supplement: Supplementary file 6 — Supplementary file6 Online Resource 4 (ESM_4) 4b Magnification of apparatus for detection of balloon surface UV365nm light. (PPTX 1083 kb) [file 13239_2023_668_MOESM6_ESM.pptx]

## Slide 1
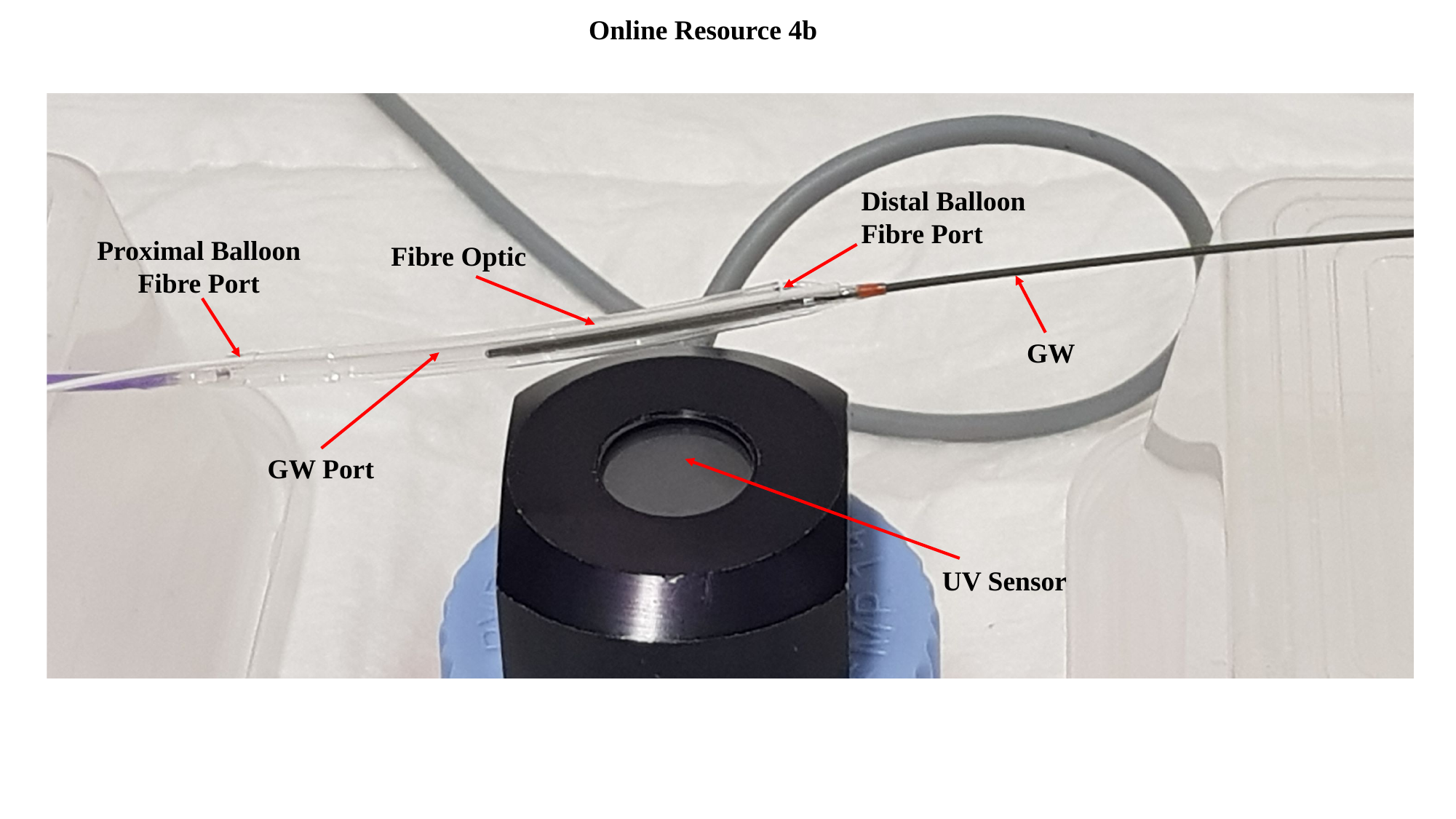

Online Resource 4b
#
Distal Balloon Fibre Port
Proximal Balloon Fibre Port
Fibre Optic
GW
GW Port
UV Sensor
