# Supplementary material for: Advances in Design and Development of Lumi-Solve: A Novel Drug-Eluting Photo-Angioplasty Device
Source: Cardiovasc Eng Technol. 2023 May 10;14(4):605–14. doi: 10.1007/s13239-023-00668-0 (PMC10465377; doi:10.1007/s13239-023-00668-0)
Supplement: Supplementary file 8 — Supplementary file8 Online Resource 5 (ESM_5) 5a-c Apparatus for determination of parallel vs. spiral fibre-optic orientation on UV365nm light transmission. (PPTX 8704 kb) [file 13239_2023_668_MOESM8_ESM.pptx]

## Slide 1
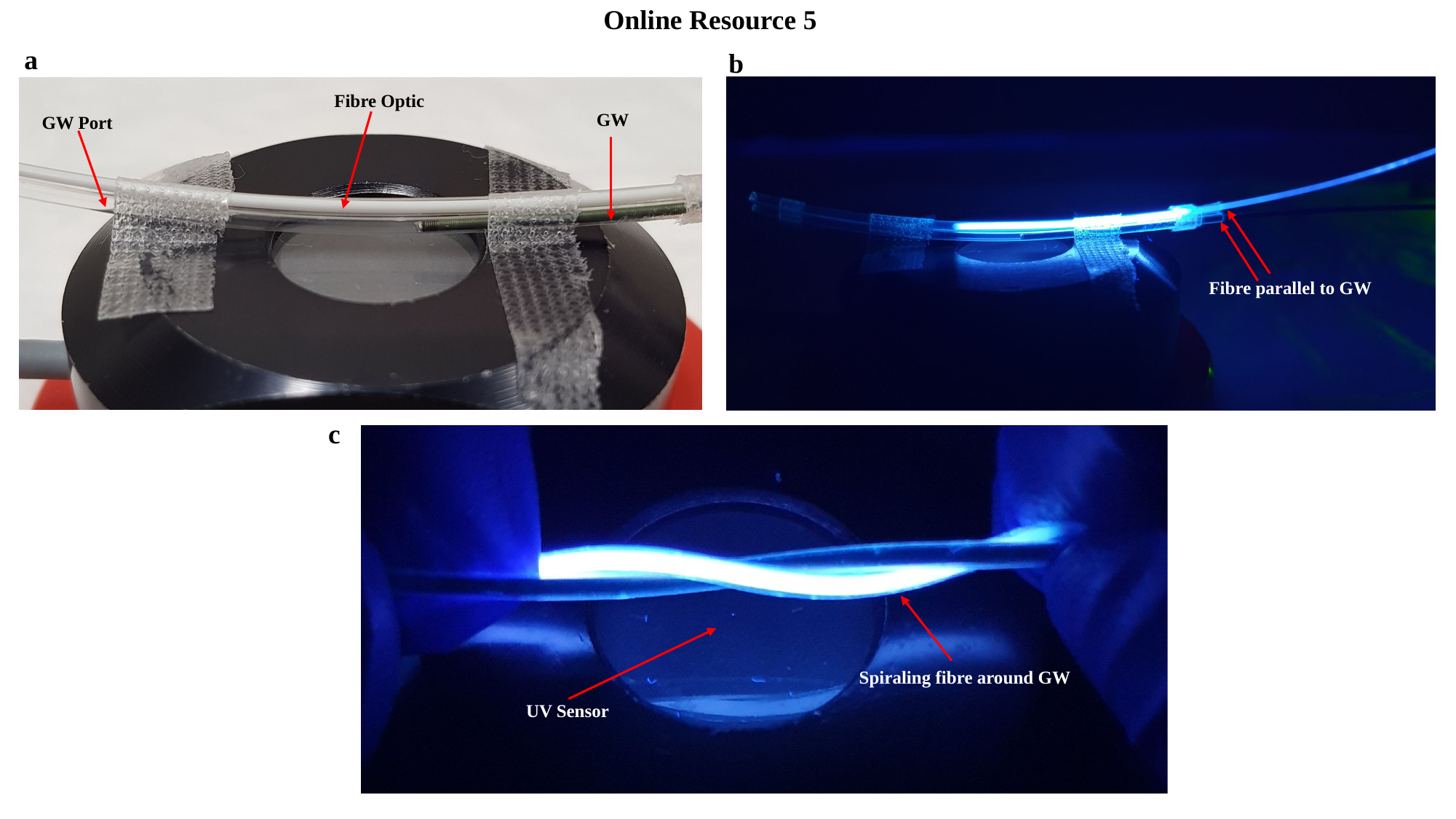

Online Resource 5
a
b
Fibre Optic
GW
GW Port
Fibre parallel to GW
c
Spiraling fibre around GW
UV Sensor
