# Supplementary material for: Advances in Design and Development of Lumi-Solve: A Novel Drug-Eluting Photo-Angioplasty Device
Source: Cardiovasc Eng Technol. 2023 May 10;14(4):605–14. doi: 10.1007/s13239-023-00668-0 (PMC10465377; doi:10.1007/s13239-023-00668-0)
Supplement: Supplementary file 9 — Supplementary file9 Online Resource 6 (ESM_6) 6a-b. Apparatus, demonstration and image GW effect on balloon surface c-MCT-3 activation. (PPTX 14238 kb) [file 13239_2023_668_MOESM9_ESM.pptx]

## Slide 1
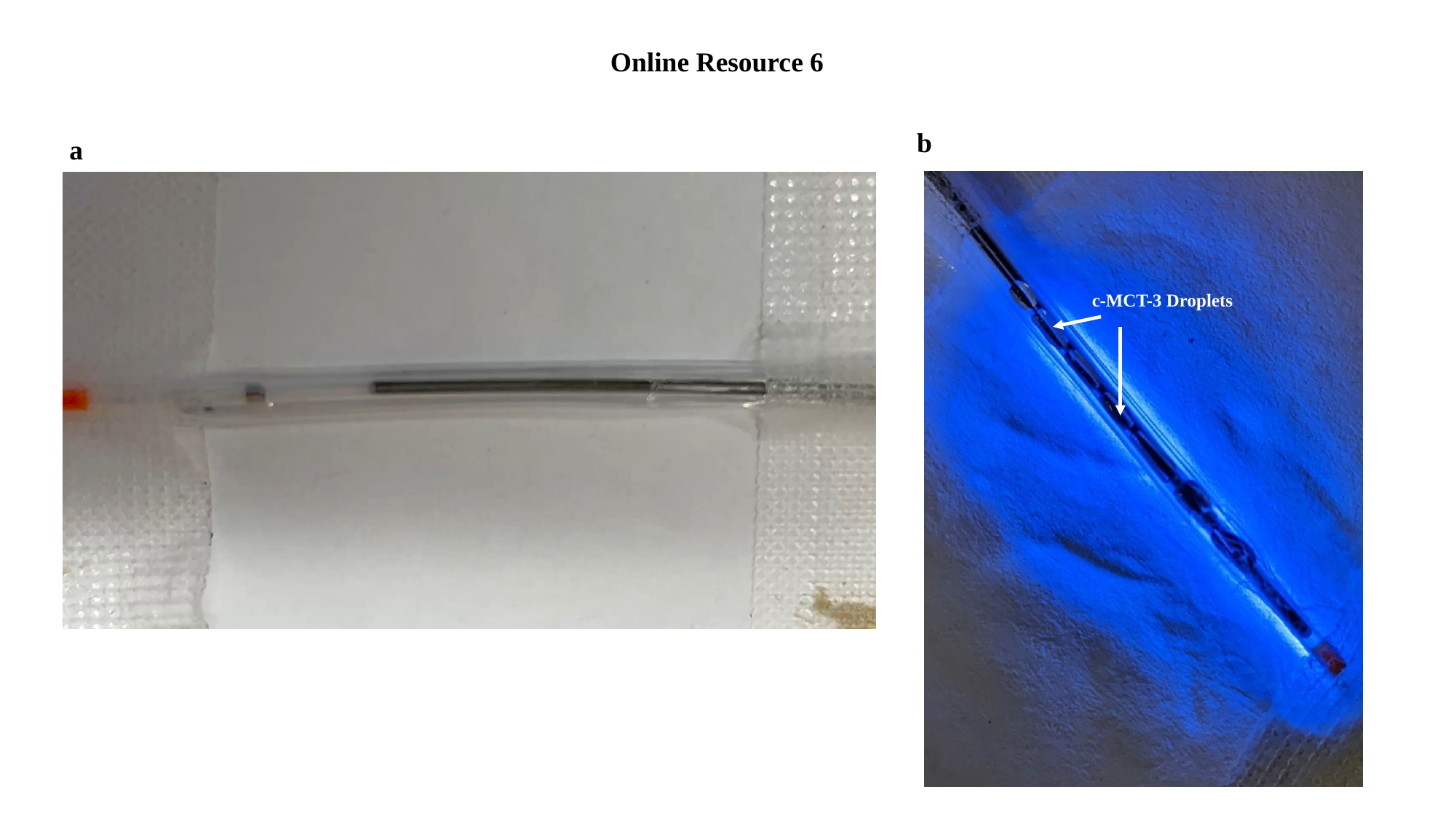

Online Resource 6
b
a
# Effects of GW on Chemical and Biological Balloon Surface Photo-MCT-3 Activation
c-MCT-3 Droplets
